# Contemporary Management of Thalassemia: A Perspective on Current Standards and the Emergence of FDA‐Approved Oral Therapy

**DOI:** 10.1002/hsr2.73178

**Published:** 2026-09-04

**Authors:** Fnu Zainab, Abdul Haseeb Hasan, Muhammad Hafi Abid, Muhammad Ali Abid

**Affiliations:** ^1^ Department of Medicine King Edward Medical University Lahore Pakistan; ^2^ Department of Research Oli Health Magazine Organization Kigali Rwanda; ^3^ Department of Internal Medicine Sentara Northern Virginia Medical Center Woodbridge Virginia USA; ^4^ Department of Medicine Faisalabad Medical University Faisalabad Pakistan

**Keywords:** ENERGIZE trial, ENERGIZE‐T trial, Mitapivat, Pyruvate kinase activation, Thalassemia

## Abstract

**Background and Aims:**

Thalassemia is an inherited hemoglobin disorder characterized by ineffective erythropoiesis, chronic anemia, and progressive multisystem complications that require lifelong management. Current treatment relies on regular red blood cell transfusions, iron chelation therapy, and supportive multidisciplinary care, while hematopoietic stem cell transplantation offers a curative option for selected patients. Although gene‐based therapies and novel pharmacological agents have expanded therapeutic options, their widespread implementation is limited by cost, accessibility, and long‐term safety considerations. This article summarizes contemporary management strategies for α and β thalassemia, with particular emphasis on the recent emergence of the FDA‐approved oral pyruvate kinase activator, mitapivat.

**Methods:**

A structured literature review was conducted using PubMed and PubMed Central. Searches included the terms “thalassemia,” “mitapivat,” “pyruvate kinase activator,” “gene therapy,” “hydroxyurea,” and “luspatercept.” Priority was given to recent systematic reviews, clinical practice guidelines, pivotal phase II and III clinical trials, regulatory publications, and peer‐reviewed studies relevant to current therapeutic practice.

**Results:**

Conventional therapies remain essential but are associated with significant limitations, including transfusion dependence, iron overload, and treatment‐related complications. Curative strategies such as hematopoietic stem cell transplantation and gene therapy are effective in selected patients but remain constrained by donor availability, toxicity, cost, and limited accessibility. Emerging pharmacological therapies target ineffective erythropoiesis and iron dysregulation. Phase III ENERGIZE and ENERGIZE‐T trials demonstrated that mitapivat significantly improved hemoglobin response in non‐transfusion‐dependent disease and reduced transfusion burden in transfusion‐dependent disease, with an acceptable safety profile, leading to FDA approval as the first oral therapy for adults with α or β thalassemia.

**Conclusion:**

Mitapivat represents a significant advance in thalassemia management by targeting erythrocyte metabolism and providing the first FDA‐approved oral treatment for anemia in adults with α or β thalassemia. Multi‐year follow‐up and real‐world studies are needed to further define its durability, safety, and role within evolving treatment strategies.

## 1 Introduction

Thalassemia is a group of inherited blood disorders caused by defective globin chain synthesis, leading to ineffective erythropoiesis, chronic anemia, and progressive multisystem complications [1]. It is particularly prevalent in the Mediterranean, Middle East, South Asia, and Southeast Asia, imposing a substantial clinical and socioeconomic burden due to lifelong treatment requirements and reduced quality of life [1]. Globally, more than 330,000 infants are born each year with clinically significant hemoglobin disorders, approximately 17% of whom are affected by thalassemia [2]. α‐Thalassemia is most commonly caused by deletions involving the α‐globin genes, whereas β‐thalassemia predominantly arises from point mutations affecting β‐globin production [3, 4, 5]. Disease severity is further influenced by genetic modifiers regulating fetal hemoglobin expression, including BCL11A, the HBS1L‐MYB intergenic region, KLF1, and ZBTB7A, contributing to considerable clinical heterogeneity [6, 7, 8].

Patients with transfusion‐dependent (TDT) thalassemia experience substantial impairment in health‐related quality of life across physical, psychological, and social domains. Poorer outcomes have been associated with iron overload, cardiac complications, hepatitis C infection, psychiatric disorders, increasing age, female sex, and adverse effects of iron chelation, highlighting the lifelong burden of disease across both adult and pediatric populations [9, 10, 11]. Current management focuses on lifelong red blood cell transfusion, iron chelation, and multidisciplinary supportive care, with hematopoietic stem cell transplantation offering a curative option for selected patients [12]. More recently, gene‐based therapies and novel pharmacological agents have expanded therapeutic options; however, limitations related to accessibility, cost, patient eligibility, long‐term safety, and durability of response continue to restrict their widespread implementation. The recent development and regulatory approval of mitapivat have introduced a novel therapeutic strategy targeting erythrocyte metabolism, warranting a critical evaluation of its role within the evolving treatment landscape of thalassemia.

## 2 Methodological Approach

This article summarizes the current evidence regarding conventional, curative, and emerging pharmacological therapies for α‐ and β‐thalassemia, with particular emphasis on mitapivat. Relevant literature was identified through structured searches of PubMed and PubMed Central using keywords including “thalassemia,” “mitapivat,” “pyruvate kinase activator,” “gene therapy,” “hydroxyurea,” and “luspatercept.” Priority was given to recent systematic reviews, clinical practice guidelines, pivotal phase II and III clinical trials, regulatory publications, and peer‐reviewed studies informing current therapeutic practice.

## 3 Conventional Therapeutic Approaches for Thalassemia

### 3.1 Blood Transfusion

Regular red blood cell transfusions constitute the cornerstone of management for patients with TDT thalassemia major, as this therapy maintains adequate hemoglobin levels, suppresses ineffective erythropoiesis, prevents severe anemia, and supports normal growth and development [13]. However, lifelong transfusion therapy inevitably results in progressive iron overload, with excess iron accumulating in the heart, liver, and endocrine organs, thereby increasing the risk of significant morbidity if left untreated [13]. Furthermore, repeated transfusions predispose patients to alloimmunization through the development of antibodies against donor red blood cell antigens, which may complicate subsequent transfusions by limiting the availability of compatible blood products and increasing transfusion‐related challenges [14]. Although regular transfusions remain indispensable for disease management, the cumulative iron burden associated with chronic transfusion therapy necessitates the concurrent use of iron chelation therapy to prevent iron‐mediated organ damage and improve long‐term clinical outcomes [15].

### 3.2 Iron Chelation Therapies

The introduction of desferrioxamine as the first clinically effective iron chelator demonstrated that excess iron resulting from chronic transfusion therapy in patients with thalassemia could be successfully removed, establishing the foundation for modern iron chelation therapy [16]. Deferiprone, an oral iron chelator, effectively reduces iron burden in chronically transfused patients with β‐thalassemia major and offers improved treatment adherence compared with subcutaneous desferrioxamine because of its oral route of administration [17]. However, its efficacy may vary among individuals, and its use is associated with adverse effects, including arthropathy, neutropenia, and agranulocytosis [17]. In patients with TDT thalassemia whose iron overload remains inadequately controlled with standard deferiprone therapy, increasing the deferiprone dose or combining it with subcutaneous desferrioxamine has been shown to significantly reduce serum ferritin levels without increasing treatment‐related toxicity, highlighting the potential benefit of combination chelation therapy [18]. Iron chelation therapy with deferoxamine, deferiprone, and deferasirox can effectively reduce iron overload in non‐transfusion‐dependent (NTDT) thalassemia syndromes, but agents like deferasirox have limitations related to dosing, toxicity, cost, and less effective cardiac iron removal, underscoring the need for individualized protocols and careful monitoring to balance efficacy and safety [19].

### 3.3 Stem Cell Transplantation

Allogeneic hematopoietic stem cell transplantation (HSCT) remains the only widely established curative treatment for TDT β‐thalassemia [20]. Although evidence from randomized controlled trials is limited, HSCT has demonstrated excellent long‐term outcomes, particularly in younger patients with HLA‐matched sibling donors. Transplant success is strongly influenced by donor compatibility and patient risk stratification, whereas older age, alternative donor sources, and high‐risk disease are associated with increased treatment‐related mortality, graft failure, and chronic graft‐versus‐host disease [21]. By replacing ineffective erythropoiesis with normal hematopoiesis, HSCT eliminates transfusion dependence; however, conditioning regimen toxicity, graft rejection, infectious complications, and limited donor availability continue to restrict its broader application [22]. Evidence from single‐center clinical experience further demonstrates high cure rates in pediatric patients while highlighting persistent complications, including acute graft‐versus‐host disease, viral reactivation, and secondary graft failure [23]. Comparison of traditional therapies for thalassemia is given in Table 1.

**Table 1:** Comparison of traditional therapies for Thalassemia.

Therapy	Efficacy	Limitations	Accessibility
Blood transfusion	Maintains Hb, suppresses ineffective erythropoiesis, improves survival and growth [13].	Iron overload, alloimmunization, lifelong dependence [14, 24]	Requires ongoing blood‐product access and repeated transfusion visits; blood‐demand/supply gaps can be a challenge [25].
Iron chelation therapy	Removes excess iron and helps prevent iron‐mediated organ damage [15].	Lifelong adherence, drug‐related toxicity, and need for regular monitoring [26]	Depends on sustained access to chelators and monitoring resources; adherence is a major practical barrier [27].
Stem cell transplantation	The only established curative option, long‐term thalassemia‐free survival is achievable in selected patients [20].	Restricted donor availability, conditioning toxicity, graft failure, GVHD, and infections [22, 23]	Limited to specialized transplant centers and eligible patients with suitable donors [28].

Abbreviations: GVHD, graft versus host disease; Hb, hemoglobin.

### 3.4 Possible Genetic Treatment Options

Gene therapy using retroviral and lentiviral vectors has demonstrated proof of concept for curing β‐thalassemia by introducing functional globin genes into autologous hematopoietic stem cells; however, concerns remain regarding insertional dysregulation of cellular genes and the potential for insertional mutagenesis, necessitating long‐term safety monitoring [29]. Building on this strategy, autologous transplantation of genetically modified hematopoietic stem cells has shown promising clinical outcomes by restoring effective hemoglobin production and reducing transfusion requirements while avoiding graft‐versus‐host disease, although insertional oncogenesis, conditioning‐related toxicity, and variable therapeutic responses continue to present important challenges [30]. More recently, genome‐editing approaches such as CRISPR/Cas9 have emerged as targeted strategies for correcting β‐thalassemia mutations or reactivating fetal hemoglobin and have shown encouraging preclinical and early clinical results; nevertheless, off‐target effects, limited delivery efficiency, and unresolved long‐term safety concerns remain significant barriers [31]. In addition, CRISPR/Cas‐edited induced pluripotent stem cells have demonstrated the potential to generate healthy erythroid cells with reduced immunological risk, although unresolved safety concerns and limited clinical validation continue to restrict their translation into routine clinical practice [32]. Despite these advances, gene‐based therapies remain limited by the need for myeloablative conditioning, complex manufacturing processes, high treatment costs, restricted accessibility, and limited long‐term safety data [33]. Their widespread clinical implementation is further hindered by uncertain long‐term durability, reimbursement challenges, and persistent barriers to equitable patient access [34].

### 3.5 Pharmacological Treatment Options

Currently, pharmacological therapies for thalassemia target distinct pathogenic mechanisms and can be broadly categorized into fetal hemoglobin inducers and erythroid maturation agents, further elaborated in Table 2.

**Table 2:** Classes of pharmacological therapy options for the treatment of thalassemia.

Drug class	Example	Mode of action	Clinical outcomes	Limitations	Common side effects
Fetal Hemoglobin Inducers	Hydroxyurea	Stimulates γ‐globin (fetal hemoglobin) production, reducing ineffective erythropoiesis [35].	Increases hemoglobin levels and reduces transfusion requirements in some patients [35, 36].	Variable response (genotype‐dependent); long‐term safety not fully established [36].	Mild myelosuppression, gastrointestinal discomfort, skin/mucosal changes [37].
Erythroid Maturation Agents	Luspatercept	Binds TGF‐β superfamily ligands, enhancing late‐stage erythroid maturation and improving RBC production [38, 39].	Reduces transfusion burden; increases hemoglobin in transfusion‐dependent and non‐transfusion‐dependent patients [38, 39]	Heterogeneous response; requires ongoing long‐term monitoring [40, 41].	Bone pain, arthralgia, headache, myalgia, dizziness, hyperuricemia [38, 39, 41].

Abbreviations: RBC, red blood cell; TGF‐β, transforming growth factor‐beta.

Emerging pharmacological therapies for β‐thalassemia aim to address the underlying pathophysiological mechanisms of the disease beyond conventional transfusion and iron chelation therapy [42]. Erythroid maturation agents such as luspatercept (ACE‐536) and sotatercept (ACE‐011) act as transforming growth factor‐β (TGF‐β) superfamily ligand traps to reduce ineffective erythropoiesis and enhance red blood cell production [41, 43]. Although both agents promote late‐stage erythropoiesis, luspatercept has progressed to phase 3 clinical trials and is FDA‐approved for TDT β‐thalassemia, whereas sotatercept demonstrated promising efficacy in phase 2 studies but has not advanced to later‐stage clinical development [41, 43]. JAK2 inhibitors such as ruxolitinib have also been investigated in phase 2a studies for their potential to modulate abnormal erythroid proliferation [44]. Similarly, hepcidin‐based therapies seek to restore iron homeostasis by increasing hepcidin activity, thereby limiting intestinal iron absorption and improving ineffective erythropoiesis in preclinical β‐thalassemia models [45]. In addition, inhibition of transmembrane serine protease 6 (TMPRSS6) using antisense oligonucleotides or small interfering RNA has shown encouraging preclinical results; however, clinical evidence remains limited, and further studies are needed to establish long‐term efficacy and safety [46]. Ferroportin inhibitors represent another promising strategy by restricting iron export and improving iron homeostasis in β‐thalassemia models [47]. Collectively, these emerging therapies have expanded the therapeutic landscape of β‐thalassemia by targeting ineffective erythropoiesis and iron dysregulation through diverse mechanisms, but further clinical validation, long‐term safety assessment, and comparative studies are still required before widespread routine use [48]. Comparison of emerging pharmacological therapies for β‐thalassemia is given in Table 3.

**Table 3:** Comparison of emerging pharmacological therapies for β‐thalassemia.

Therapy	Mechanism	Efficacy	Limitation
Luspatercept	TGF‐β superfamily ligand trap that enhances late‐stage erythropoiesis [49].	Reduces transfusion burden in transfusion‐dependent β‐thalassemia and improves hemoglobin/erythropoiesis [50].	Benefit is mainly established in adult TDT/selected NTDT populations; treatment is not curative and requires continued dosing and monitoring [51].
Sotatercept	Activin receptor/TGF‐β ligand trap that promotes late‐stage erythropoiesis [43].	In a phase II study, it increased hemoglobin and reduced transfusion burden in some patients [43].	Evidence is limited to early‐phase clinical data; small sample size and short follow‐up limit conclusions [43].
Ruxolitinib	JAK1/JAK2 inhibitor that modulates signaling involved in abnormal erythroid proliferation [44].	Showed a slight rise in pretransfusion hemoglobin and a clear reduction in spleen size in transfused thalassemia patients [52].	Clinical benefit was modest, with limited effect on transfusion requirements; no further phase 3 development was planned in that setting [44].
Hepcidin‐based therapies	Increase hepcidin activity to restrict iron absorption and iron availability [45].	Improved anemia and iron overload in β‐thalassemic mouse models [53].	Evidence is primarily preclinical, so human efficacy and long‐term safety remain uncertain [53].
TMPRSS6 inhibitors	Suppress TMPRSS6 to increase hepcidin and improve iron homeostasis [46].	Preclinical TMPRSS6 suppression has shown benefit for erythropoiesis/iron regulation in development programs [46].	Clinical evidence remains limited, and long‐term human efficacy and safety are still being established [46].
Ferroportin inhibitors	Reduce iron export by inhibiting ferroportin [47].	Improved ineffective erythropoiesis and iron homeostasis in a β‐thalassemia model [47].	Evidence is still preclinical, with human clinical data not yet established [47].

Abbreviations: JAK, Janus Kinase; NTDT, Non‐Transfusion Dependent Thalassemia; TDT, Transfusion Dependent Thalassemia; TGF‐β, Transforming Growth Factor Beta; TMPRSS6, Transmembrane Protease, Serine 6.

### 3.6 Pyruvate Kinase Activator Mitapivat

Mitapivat functions by activating erythroid pyruvate kinase isoforms and metabolically reprogramming human β‐thalassemic erythroblasts, thereby improving erythroblast maturation, survival, and resistance to oxidative damage [54]. In a β‐thalassemia mouse model, mitapivat reduced hemolysis, improved anemia, enhanced red blood cell survival, ameliorated ineffective erythropoiesis, and favorably modulated iron homeostasis and oxidative stress, providing the rationale for clinical development [55]. In an open‐label phase 2 study, long‐term mitapivat treatment produced sustained hemoglobin increases and improvements in hemolysis and ineffective erythropoiesis in adults with NTDT α‐ or β‐thalassemia, with an acceptable safety profile over extended follow‐up [56]. ENERGIZE and ENERGIZE‐T were randomized, double‐blind, placebo‐controlled phase 3 studies in adults with α‐ or β‐thalassemia; ENERGIZE assessed hemoglobin response in NTDT disease, whereas ENERGIZE‐T assessed transfusion reduction in TDT disease [57, 58]. On the basis of these data, the FDA approved mitapivat (Aqvesme) for anemia in adults with α‐ or β‐thalassemia, making it the first oral treatment option for β‐thalassemia and the first approved drug for α‐thalassemia; access is restricted through a REMS program because of liver toxicity observed in clinical trials [58].

Within the current treatment landscape, mitapivat occupies a distinct clinical role as an oral, disease‐modifying agent that targets erythrocyte metabolism rather than replacing established curative or transfusion‐based strategies. Unlike regular blood transfusion, which remains the mainstay for severely affected patients but carries lifelong risks of iron overload and alloimmunization, mitapivat lowers transfusion burden without the logistical demands of transfusion therapy [58]. Relative to luspatercept, which is administered subcutaneously and acts on late‐stage erythroid maturation, mitapivat is taken orally and acts earlier in the erythropoietic pathway through direct pyruvate kinase activation. Compared with HSCT and gene therapy, which remain the only potentially curative options but are limited by donor availability, conditioning‐related toxicity, and high cost, mitapivat offers a lower‐risk, non‐curative alternative for patients who are ineligible for or wish to defer curative procedures. Mitapivat is therefore best positioned as an adjunctive or alternative oral option within a stepwise, individualized treatment approach, complementing rather than supplanting existing conventional and curative therapies.

### 3.7 ENERGIZE and ENERGIZE‐T Trials

The phase 3 ENERGIZE trial was an international, randomized, double‐blind, placebo‐controlled study that evaluated the efficacy and safety of mitapivat in adults with NTDT α‐ or β‐thalassemia [59]. A total of 194 participants were randomized to receive mitapivat (*n* = 130) or placebo (*n* = 64). The primary endpoint, defined as a sustained hemoglobin response of ≥ 1.0 g/dL increase from baseline, was achieved by 55 of 130 patients (42.3%) receiving mitapivat compared with 1 of 64 patients (1.6%) receiving placebo, corresponding to a least‐squares mean difference of 41% (95% CI, 32–50; *p* < 0.0001) [59]. Improvements were also observed in patient‐reported fatigue outcomes, while mitapivat was generally well tolerated, with an adverse event profile broadly comparable to placebo [59]. These findings demonstrate that mitapivat provides clinically meaningful improvements in hemoglobin levels in adults with NTDT thalassemia.

The phase 3 ENERGIZE‐T trial was a global, randomized, double‐blind, placebo‐controlled study designed to evaluate mitapivat in adults with TDT α‐ or β‐thalassemia [60]. A total of 258 patients were randomized in a 2:1 ratio to receive mitapivat (*n* = 171) or placebo (*n* = 87) for 48 weeks. The primary endpoint, transfusion reduction response (TRR), defined as a ≥ 50% reduction in transfused red blood cell units with a reduction of at least two units during any consecutive 12‐week period, was achieved in 30.4% of patients receiving mitapivat compared with 12.6% of those receiving placebo (*p* = 0.0003) [60]. Mitapivat also demonstrated superiority across key secondary endpoints, including a ≥ 50% reduction in transfusion burden over any 24‐week period (13.5% vs. 2.3%, *p* = 0.0003), a ≥ 33% reduction in transfusion burden during Weeks 13–48 (14.6% vs. 1.1%, *p* < 0.0001), and a ≥ 50% reduction during Weeks 13–48 (7.6% vs. 1.1%, *p* = 0.0056). Furthermore, transfusion independence was achieved in 9.9% of patients receiving mitapivat compared with 1.1% of those receiving placebo [60]. Treatment was generally well tolerated, with low discontinuation rates due to adverse events, supporting the efficacy and acceptable safety profile of mitapivat in TDT thalassemia.

Collectively, the ENERGIZE and ENERGIZE‐T trials demonstrated clinically meaningful improvements in hemoglobin response and transfusion burden, respectively, while maintaining an acceptable safety profile, supporting mitapivat as the first approved oral pyruvate kinase activator for adults with both NTDT and TDT α‐ or β‐thalassemia.

### 3.8 Limitations in Trials

Despite the promising efficacy and safety demonstrated in the ENERGIZE and ENERGIZE‐T trials, several limitations should be considered. The 24‐week double‐blind treatment period in ENERGIZE limits assessment of the long‐term durability of hemoglobin response, disease progression, and extended safety outcomes [59]. Although efficacy was generally consistent across prespecified subgroups, additional data are required to better define treatment responses across different genotypes, age groups, racial and ethnic populations, and baseline disease characteristics [59]. Similarly, the 48‐week duration of ENERGIZE‐T limits evaluation of the long‐term durability of transfusion reduction and the potential effects of mitapivat on iron overload, organ function, and late safety outcomes [60]. Furthermore, both trials primarily evaluated hematologic endpoints, while longer‐term functional outcomes, health‐related quality of life, and disease‐related complications require further investigation [61]. Finally, the strict eligibility criteria inherent to randomized clinical trials may limit the generalizability of these findings to broader real‐world thalassemia populations. Consequently, long‐term extension studies and real‐world evidence will be essential to further define the durability, safety, and effectiveness of mitapivat across diverse patient populations. A minimum of 2–3 years of continuous follow‐up is generally needed to adequately assess the durability of hemoglobin and transfusion responses, while follow‐up of 5 years or longer is necessary to meaningfully evaluate cumulative long‐term safety, including hepatic, iron‐related, and other organ‐specific outcomes. Published experience with other thalassemia therapies provides a useful benchmark. The pivotal phase 2 extension study of luspatercept followed patients for up to 5 years, while the phase 3 BELIEVE trial reported efficacy and safety after approximately 3 years of follow‐up [51, 62]. These findings suggest that a comparable multi‐year follow‐up of mitapivat is needed to establish its long‐term durability and safety.

### 3.9 Future Perspective

The completion of the phase 3 ENERGIZE and ENERGIZE‐T trials, together with the recent U.S. Food and Drug Administration approval of Aqvesme (mitapivat) as the first FDA‐approved oral treatment for anemia in adults with α‐ or β‐thalassemia, marks an important milestone in the management of thalassemia. Multi‐year follow‐up from ongoing extension studies and post‐marketing surveillance will be essential to determine the durability of the improvements in hemoglobin response and transfusion burden observed in these trials, as well as their effects on clinically meaningful outcomes such as iron overload, end‐organ complications, and overall survival. In addition, real‐world evidence will be crucial to further characterize the safety and effectiveness of mitapivat across broader and more diverse patient populations, including differences related to age, race, genotype, baseline disease severity, and coexisting medical conditions that may not have been fully represented in clinical trials. Future studies should also evaluate the role of mitapivat in pediatric populations, earlier intervention during the disease course, and combination strategies with other emerging therapies that target ineffective erythropoiesis or iron dysregulation. Furthermore, incorporation of patient‐reported outcomes and health‐related quality‐of‐life measures into future clinical studies will be important to better define the overall clinical value of oral therapy beyond traditional hematologic endpoints. As the first FDA‐approved oral agent for anemia in thalassemia, mitapivat establishes proof of concept for targeting erythrocyte metabolism and may facilitate the development of future metabolic therapies for hereditary hemoglobinopathies.

## 4 Conclusion

Thalassemia remains a lifelong disorder with substantial unmet clinical needs despite advances in supportive care and curative therapies for selected patients. Mitapivat, through pyruvate kinase activation, represents a significant therapeutic advance by targeting the underlying metabolic dysfunction that contributes to ineffective erythropoiesis. The positive outcomes of the phase 3 ENERGIZE and ENERGIZE‐T trials led to the FDA approval of Aqvesme™ (mitapivat) for the treatment of anemia in adults with α‐ or β‐thalassemia, establishing the first approved oral therapy for β‐thalassemia and the first approved pharmacological treatment for α‐thalassemia. Although continued multi‐year follow‐up and real‐world studies are needed to further define the durability of response, long‐term safety, and effectiveness across diverse patient populations, mitapivat represents a major step toward precision, disease‐modifying therapy, and has the potential to reshape the future management of hereditary hemoglobinopathies.

## Author Contributions

**FNU Zainab:** conceptualization, writing – original draft, writing – review and editing. **Abdul Haseeb Hasan:** conceptualization, writing – original draft, writing – review and editing. **Muhammad Hafi Abid:** conceptualization, writing – original draft, writing – review and editing. **Muhammad Ali Abid:** writing – original draft, writing – review and editing.

## Funding

The authors have nothing to report.

## Ethics Statement

The authors have nothing to report.

## Conflicts of Interest

The authors declare no conflicts of interest.

## Declaration of Generative AI and AI‐Assisted Technologies in the Writing Process

There is nothing to disclose.

## Guarantor

All authors have read and approved the final version of the manuscript. Dr. Abdul Haseeb Hasan had full access to all of the data in this study and takes complete responsibility for the integrity of the data and the accuracy of the data analysis.

## Transparency Statement

Dr. Abdul Haseeb Hasan affirms that this manuscript is an honest, accurate, and transparent account of the study being reported; that no important aspects of the study have been omitted; and that any discrepancies from the study as planned have been explained.

## Data Availability

Data sharing is not applicable to this article as no datasets were generated or analyzed during the current study.

## References

[1] I. Z. Sadiq , F. S. Abubakar , H. S. Usman , et al., “Thalassemia: Pathophysiology, Diagnosis, and Advances in Treatment,” Thalassemia Reports 14, no. 4 (2024): 81–102, 10.3390/thalassrep14040010.

[2] A. D. Alahmari , M. Aljurf , A. Alseraihy , et al., “Hematopoietic Stem Cell Transplantation for Patients With Sickle Cell Disease in the Eastern Mediterranean,” Hematology/Oncology and Stem Cell Therapy 13, no. 2 (2020): 106–110, 10.1016/j.hemonc.2020.02.002. 32202251

[3] N. Tesio and D. E. Bauer , “Molecular Basis and Genetic Modifiers of Thalassemia,” Hematology/Oncology Clinics of North America 37, no. 2 (2023): 273–299, 10.1016/j.hoc.2022.12.001. 36907603 PMC10122828

[4] D. R. Higgs , “The Molecular Basis of α‐Thalassemi,” Cold Spring Harbor Perspectives in Medicine 3, no. 1 (2013): a011718, 10.1101/cshperspect.a011718. 23284078 PMC3530043

[5] S. L. Thein , “The Molecular Basis of β‐Thalassemia,” Cold Spring Harbor Perspectives in Medicine 3, no. 5 (2013): a011700, 10.1101/cshperspect.a011700. 23637309 PMC3633182

[6] K. Qin , P. Huang , R. Feng , et al., “Dual Function NFI Factors Control Fetal Hemoglobin Silencing in Adult Erythroid Cells,” Nature Genetics 54, no. 6 (2022): 874–884, 10.1038/s41588-022-01076-1. 35618846 PMC9203980

[7] A. Perkins , X. Xu , D. R. Higgs , et al., “Krüppeling Erythropoiesis: An Unexpected Broad Spectrum of Human Red Blood Cell Disorders Due to KLF1 Variants,” Blood 127, no. 15 (2016): 1856–1862, 10.1182/blood-2016-01-694331. 26903544 PMC4832505

[8] G. E. Martyn , B. Wienert , L. Yang , et al., “Natural Regulatory Mutations Elevate the Fetal Globin Gene via Disruption of BCL11A or ZBTB7A Binding,” Nature Genetics 50, no. 4 (2018): 498–503, 10.1038/s41588-018-0085-0. 29610478

[9] Sh Ansari , A. Baghersalimi , A. Azarkeivan , M. Nojomi , and A. Hassanzadeh Rad , “Quality of Life in Patients With Thalassemia Major,” Iranian Journal of Pediatric Hematology and Oncology 4, no. 2 (2014): 57–63. 25002926 PMC4083201

[10] A. Sobota , R. Yamashita , Y. Xu , et al., “Quality of Life in Thalassemia: A Comparison of SF‐36 Results From the Thalassemia Longitudinal Cohort to Reported Literature and the US Norms,” American Journal of Hematology 86, no. 1 (2011): 92–95, 10.1002/ajh.21896. 21061309 PMC4250926

[11] N. Dhirar , J. Khandekar , D. Bachani , and D. Mahto , “Thalassemia Major: How Do We Improve Quality of Life?,” SpringerPlus 5, no. 1 (2016): 1895, 10.1186/s40064-016-3568-4. 27843752 PMC5084148

[12] D. Farmakis , J. Porter , A. Taher , M. Domenica Cappellini , M. Angastiniotis , and A. Eleftheriou , “2021 Thalassaemia International Federation Guidelines for the Management of Transfusion‐Dependent Thalassemia,” HemaSphere 6, no. 8 (2022): e732, 10.1097/HS9.0000000000000732. 35928543 PMC9345633

[13] M. M. A. Gamaleldin , S. M. N. S. Abdelhalim , and I. Abraham , “Recent Advances in Thalassemia Management: From Curative Therapies to Artificial Intelligence,” Thalassemia Reports 16, no. 2 (2026): 7, 10.3390/thalassrep16020007.

[14] E. Vichinsky , L. Neumayr , S. Trimble , et al., “Transfusion Complications in Thalassemia Patients: A Report From the Centers for Disease Control and Prevention (CME),” Transfusion 54, no. 4 (2014): 972–981; quiz 971, 10.1111/trf.12348. 23889533 PMC4410835

[15] P. Cianciulli , “Iron Chelation Therapy in Thalassemia Syndromes,” Mediterranean Journal of Hematology and Infectious Diseases 1, no. 1 (2009): e2009034, 10.4084/MJHID.2009.034. 21415999 PMC3033168

[16] R. M. Bannerman , S. T. Callender , and D. L. Williams , “Effect of Desferrioxamine and D.T.P.A. in Iron Overload,” BMJ 2, no. 5319 (1962): 1573–1577, 10.1136/bmj.2.5319.1573. 20789564 PMC1926807

[17] J. A. Barman Balfour and R. H. Foster , “Deferiprone,” Drugs 58, no. 3 (1999): 553–578, 10.2165/00003495-199958030-00021. 10493280

[18] Wonke, Wright, Hoffbrand, Wright, Hoffbrand ., “Combined Therapy With Deferiprone and Desferrioxamine,” British Journal of Haematology 103, no. 2 (1998): 361–364, 10.1046/j.1365-2141.1998.01002.x. 9827905

[19] C. N. Kontoghiorghe and G. J. Kontoghiorghes , “Efficacy and Safety of Iron‐Chelation Therapy With Deferoxamine, Deferiprone, and Deferasirox for the Treatment of Iron‐Loaded Patients With Non‐Transfusion‐Dependent Thalassemia Syndromes,” Drug Design, Development and Therapy 10 (2016): 465–481, 10.2147/DDDT.S79458. 26893541 PMC4745840

[20] A. Sharma , V. A. Jagannath , and L. Puri , “Hematopoietic Stem Cell Transplantation for People With β‐Thalassaemia,” Cochrane Database of Systematic Reviews 2021, no. 4 (2021): CD008708, 10.1002/14651858.CD008708.pub5. PMC8078520 33880750

[21] E. Angelucci , S. Matthes‐Martin , D. Baronciani , et al., “Hematopoietic Stem Cell Transplantation in Thalassemia Major and Sickle Cell Disease: Indications and Management Recommendations From an International Expert Panel,” Haematologica 99, no. 5 (2014): 811–820, 10.3324/haematol.2013.099747. 24790059 PMC4008115

[22] E. Angelucci , F. Pilo , C. Targhetta , et al., “Hematopietic Stem Cell Transplantation in Thalassemia and Related Disorders,” Mediterranean Journal of Hematology and Infectious Diseases 1, no. 1 (2009): e2009015, 10.4084/MJHID.2009.015. 21415993 PMC3033161

[23] M. Mustafa , M. Qatawneh , M. Jazazi , et al., “Hematopoietic Stem Cell Transplantation in Thalassemia Patients: A Jordanian Single Centre Experience,” Materia Socio Medica 32, no. 4 (2020): 277–282, 10.5455/msm.2020.32.277-282. 33628130 PMC7879431

[24] A. A. Thompson , M. J. Cunningham , S. T. Singer , et al., “Red Cell Alloimmunization in a Diverse Population of Transfused Patients With Thalassaemia,” British Journal of Haematology 153, no. 1 (2011): 121–128, 10.1111/j.1365-2141.2011.08576.x. 21323889 PMC5728106

[25] M. H. Albagshi , M. Saad , A. M. Aljassem , et al., “Blood Demand and Challenges for Patients With Beta‐Thalassemia Major in Eastern Saudi Arabia,” Cureus 13, no. 8 (2021): e17470, 10.7759/cureus.17470. 34603865 PMC8475924

[26] S. Entezari , S. M. Haghi , N. Norouzkhani , et al., “Iron Chelators in Treatment of Iron Overload,” Journal of Toxicology 2022 (2022): 4911205, 10.1155/2022/4911205. 35571382 PMC9098311

[27] L. E. Wang , S. Muttar , and S. M. Badawy , “The Challenges of Iron Chelation Therapy in Thalassemia: How Do We Overcome Them?,” Expert Review of Hematology 18, no. 5 (2025): 351–357, 10.1080/17474086.2025.2489562. 40181584 PMC12125653

[28] B. Modell , “Global Epidemiology of Haemoglobin Disorders and Derived Service Indicators,” Bulletin of the World Health Organization 2008, no. 6 (2008): 480–487, 10.2471/blt.06.036673. PMC2647473 18568278

[29] J. Malay , R. A. A. Salama , G. S. Alam Qureshi , et al., “Gene Therapy: A Revolutionary Step in Treating Thalassemia,” Hematology Reports 16, no. 4 (2024): 656–668, 10.3390/hematolrep16040064. 39449307 PMC11503351

[30] G. Karponi and N. Zogas , “Gene Therapy For Beta‐Thalassemia: Updated Perspectives,” Application of Clinical Genetics 12 (2019): 167–180, 10.2147/TACG.S178546. 31576160 PMC6765258

[31] H. Liu and P. Zhang , “Advances in β‐Thalassemia Gene Therapy: CRISPR/Cas Systems and Delivery Innovations,” Cells 14, no. 20 (2025): 1595, 10.3390/cells14201595. 41148810 PMC12562837

[32] J. Shu , X. Xie , S. Wang , et al., “Crispr/Cas‐Edited Ipscs and Mesenchymal Stem Cells: A Concise Review of Their Potential in Thalassemia Therapy,” Frontiers in Cell and Developmental Biology 13 (2025): 1595897, 10.3389/fcell.2025.1595897. 40970094 PMC12441039

[33] A. N. Cheng and J. L. Kwiatkowski , “Current Therapeutic Landscape of β‐Thalassemia: Focus on Gene Therapy,” Therapeutic Advances in Rare Disease 7 (2026): 26330040261433028, 10.1177/26330040261433028. 41919270 PMC13033867

[34] S. Phares , M. Trusheim , S. K. Emond , and S. D. Pearson , “Managing the Challenges of Paying for Gene Therapy: Strategies for Market Action and Policy Reform in the United States,” Journal of Comparative Effectiveness Research 13, no. 12 (2024): e240118, 10.57264/cer-2024-0118. 39540549 PMC11609966

[35] N. Yasara , N. Wickramarathne , C. Mettananda , et al., “A Randomised Double‐Blind Placebo‐Controlled Clinical Trial of Oral Hydroxyurea for Transfusion‐Dependent Β‐Thalassaemia,” Scientific Reports 12 (2022): 2752, 10.1038/s41598-022-06774-8. 35177777 PMC8854735

[36] M. I. Hatamleh , V. S. H. Chenna , H. Contractor , et al., “Efficacy of Hydroxyurea in Transfusion‐Dependent Major β‐Thalassemia Patients: A Meta‐Analysis,” Cureus 15, no. 4 (2023): e38135, 10.7759/cureus.38135. 37252463 PMC10213992

[37] A. Ghasemi , B. Keikhaei , and R. Ghodsi , “Side Effects of Hydroxyurea in Patients With Thalassemia Major and Thalassemia Intermedia and Sickle Cell Anemia,” Iranian Journal of Pediatric Hematology and Oncology 4, no. 3 (2014): 114–117. 25254090 PMC4173031

[38] I. M. Dighriri , K. K. Alrabghi , D. M. Sulaiman , et al., “Efficacy and Safety of Luspatercept in the Treatment of β‐Thalassemia: A Systematic Review,” Cureus 14, no. 11 (2022): e31570, 10.7759/cureus.31570. 36540460 PMC9756914

[39] M. D. Cappellini and A. T. Taher , “The Use of Luspatercept for Thalassemia in Adults,” Blood Advances 5, no. 1 (2021): 326–333, 10.1182/bloodadvances.2020002725. 33570654 PMC7805339

[40] F. Longo , I. Motta , V. Pinto , et al., “Treating Thalassemia Patients With Luspatercept: An Expert Opinion Based on Current Evidence,” Journal of Clinical Medicine 12, no. 7 (2023): 2584, 10.3390/jcm12072584. 37048666 PMC10094868

[41] A. Piga , F. Longo , M. R. Gamberini , et al., “Long‐Term Safety and Erythroid Response With Luspatercept Treatment in Patients With β‐Thalassemia,” Therapeutic Advances in Hematology 13 (2022): 20406207221134404, 10.1177/20406207221134404. 36505885 PMC9726852

[42] I. Motta , R. Bou‐Fakhredin , A. T. Taher , and M. D. Cappellini , “Beta Thalassemia: New Therapeutic Options Beyond Transfusion and Iron Chelation,” Drugs 80, no. 11 (2020): 1053–1063, 10.1007/s40265-020-01341-9. 32557398 PMC7299245

[43] M. D. Cappellini , J. Porter , R. Origa , et al., “Sotatercept, a Novel Transforming Growth Factor β Ligand Trap, Improves Anemia in β‐Thalassemia: A Phase II, Open‐Label, Dose‐Finding Study,” Haematologica 104, no. 3 (2019): 477–484, 10.3324/haematol.2018.198887. 30337358 PMC6395345

[44] A. T. Taher , Z. Karakas , E. Cassinerio , et al., “Efficacy and Safety of Ruxolitinib in Regularly Transfused Patients With Thalassemia: Results From a Phase 2a Study,” Blood 131, no. 2 (2018): 263–265, 10.1182/blood-2017-06-790121. 29097381 PMC5757682

[45] S. Gardenghi , P. Ramos , M. F. Marongiu , et al., “Hepcidin as a Therapeutic Tool to Limit Iron Overload and Improve Anemia in β‐Thalassemic Mice,” Journal of Clinical Investigation 120, no. 12 (2010): 4466–4477, 10.1172/JCI41717. 21099112 PMC2993583

[46] T. Ganz , E. Nemeth , S. Rivella , et al., “TMPRSS6 as a Therapeutic Target for Disorders of Erythropoiesis and Iron Homeostasis,” Advances in Therapy 40, no. 4 (2023): 1317–1333, 10.1007/s12325-022-02421-w. 36690839 PMC10070284

[47] V. Manolova , N. Nyffenegger , A. Flace , et al., “Oral Ferroportin Inhibitor Ameliorates Ineffective Erythropoiesis in a Model of β‐Thalassemia,” Journal of Clinical Investigation 130, no. 1 (2020): 491–506, 10.1172/JCI129382. PMC6934209 31638596

[48] R. Bou‐Fakhredin , I. Motta , and M. D. Cappellini , “Advancing the Care of β‐Thalassaemia Patients With Novel Therapies,” Blood Transfusion 20, no. 1 (2022): 78–88, 10.2450/2021.0265-21. 34694225 PMC8796844

[49] K. M. Musallam , S. Sheth , M. D. Cappellini , A. Kattamis , K. H. M. Kuo , and A. T. Taher , “Luspatercept for Transfusion‐Dependent β‐Thalassemia: Time to Get Real,” Therapeutic Advances in Hematology 14 (2023): 20406207231195594, 10.1177/20406207231195594. 37645382 PMC10460678

[50] S. Sheth , A. T. Taher , T. D. Coates , A. Kattamis , and M. D. Cappellini , “Management of Luspatercept Therapy in Patients With Transfusion‐Dependent β‐Thalassaemia,” British Journal of Haematology 201, no. 5 (2023): 824–831, 10.1111/bjh.18801. 37037668

[51] Y. Aydinok , “Highlights on the Luspatercept Treatment in Thalassemia,” Thalassemia Reports 13, no. 1 (2023): 77–84, 10.3390/thalassrep13010008.

[52] C. Casu , V. L. Presti , P. R. Oikonomidou , et al., “Short‐Term Administration of JAK2 Inhibitors Reduces Splenomegaly in Mouse Models of β‐Thalassemia Intermedia and Major,” Haematologica 103, no. 2 (2018): e46–e49, 10.3324/haematol.2017.181511. 29097498 PMC5792284

[53] M. Chappell and S. Rivella , “New Potential Players in Hepcidin Regulation,” Haematologica 104, no. 9 (2019): 1691–1693, 10.3324/haematol.2019.224311. 31473605 PMC6717596

[54] A. Siciliano , A. D'Alessandro , A. Matte , et al., “Mitapivat Metabolically Reprograms Human β‐Thalassemic Erythroblasts, Increasing Their Responsiveness to Oxidation,” Blood Advances 9, no. 11 (2025): 2818–2830, 10.1182/bloodadvances.2024013591. 40085946 PMC12167806

[55] A. Matte , E. Federti , C. Kung , et al., “The Pyruvate Kinase Activator Mitapivat Reduces Hemolysis and Improves Anemia in a β‐Thalassemia Mouse Model,” Journal of Clinical Investigation 131, no. 10 (2021): e144206, 10.1172/JCI144206. 33822774 PMC8121526

[56] K. H. M. Kuo , D. M. Layton , A. Lal , et al., “P1522: Long‐Term Efficacy and Safety of the Oral Pyruvate Kinase Activator Mitapivat in Adults With Non–Transfusion‐Dependent Alpha‐ or Beta‐Thalassemia,” HemaSphere 6, no. Suppl (2022): 1403–1404, 10.1097/01.HS9.0000848944.56958.95.

[57] K. Kuo , D. Layton , H. Al‐Samkari , et al., “P112: Energize and Energize‐T: Two Phase 3, Randomized, Double‐Blind, Placebo‐Controlled Studies of Mitapivat in Adults With Non‐Transfusion‐Dependent or Transfusion‐Dependent Alpha‐ or Beta‐Thalassemia,” HemaSphere 6, no. Suppl (2022): 23–24, 10.1097/01.HS9.0000821540.81071.fa.

[58] A. Kattamis , K. Bistas , V. Mitros , E. F. Agiomavriti Stefanopoulou , and P. Delaporta , “Evaluating Mitapivat for the Treatment of Alpha or Beta Thalassemia,” Expert Opinion on Pharmacotherapy 27, no. 11 (2026): 1045–1052, 10.1080/14656566.2026.2705347. 42472450

[59] A. T. Taher , H. Al‐Samkari , Y. Aydinok , et al., “Mitapivat in Adults With Non‐Transfusion‐Dependent α‐Thalassaemia or β‐Thalassaemia (ENERGIZE): A Phase 3, International, Randomised, Double‐Blind, Placebo‐Controlled Trial,” Lancet 406, no. 10498 (2025): 33–42, 10.1016/S0140-6736(25)00635-X. 40544857

[60] M. D. Cappellini , S. Sheth , A. T. Taher , et al., “ENERGIZE‐T: A Global, Phase 3, Double‐Blind, Randomized, Placebo‐Controlled Study of Mitapivat in Adults With Transfusion‐Dependent Alpha‐ or Beta‐Thalassemia,” Blood 144, no. Suppl 1 (2024): 409, 10.1182/blood-2024-200867.

[61] K. Kuo , D. Layton , H. Al‐Samkari , et al., “ENERGIZE and ENERGIZE‐T: Two Phase 3, Randomized, Double‐Blind, Placebo‐Controlled Studies of Mitapivat in Adults With Non‐Transfusion‐Dependent or Transfusion‐Dependent Alpha‐ or Beta‐Thalassemia,” Hematology, Transfusion and Cell Therapy 43 (2021): S20, 10.1016/j.htct.2021.10.035.

[62] M. D. Cappellini , V. Viprakasit , P. Georgiev , et al., “Long‐Term Efficacy and Safety of Luspatercept for the Treatment of Anaemia in Patients With Transfusion‐Dependent β‐Thalassaemia (BELIEVE): Final Results From a Phase 3 Randomised Trial,” Lancet Haematology 12, no. 3 (2025): e180–e189, 10.1016/S2352-3026(24)00376-4. 39947215

